# Simulation Training in Neuroangiography: Transfer to Reality

**DOI:** 10.1007/s00270-020-02479-5

**Published:** 2020-05-11

**Authors:** Kornelia Kreiser, Kim G. Gehling, Lea Ströber, Claus Zimmer, Jan S. Kirschke

**Affiliations:** Department of Diagnostic and Interventional Neuroradiology, Klinikum rechts der Isar, Technische Universität München, Munich, Germany

**Keywords:** Patient-specific rehearsal, Neuroangiography, Silent ischemia, Training, Novices

## Abstract

**Purpose:**

Endovascular simulation is an established and validated training method, but there is still no proof of direct patient’s benefit, defined as lower complication rate. In this study, the impact of such a training was investigated for rehearsal of patient-specific cases as well as for a structured simulation curriculum to teach angiographer novices.

**Materials and Methods:**

A total of 40 patients undergoing a diagnostic neuroangiography were randomized in a training and control group. In all training group patients, the angiographer received a patient-anatomy-specific rehearsal on a high-fidelity simulator prior to the real angiography. Radiation exposure, total duration, fluoroscopy time and amount of contrast agent of the real angiography were recorded. Silent cerebral ischemia was counted by magnetic resonance diffusion-weighted imaging (DWI). Additionally, the first 30 diagnostic neuroangiographies of six novices were compared (*n*_total_ = 180). Three novices had undergone a structured simulation curriculum; three had acquired angiographic skills without simulation.

**Results:**

No differences were found in the number of DWI lesions or in other quality measures of the angiographies performed with and without patient-specific rehearsal. A structured simulation curriculum for angiographer novices reduced fluoroscopy time significantly and radiation exposure. The curriculum had no influence on the total duration of the examination, the amount of contrast medium or the number of catheters used.

**Conclusion:**

There was no measurable benefit of patient-anatomy-specific rehearsal for an unselected patient cohort. A structured simulation-based curriculum to teach angiographic skills resulted in a reduction of fluoroscopy time and radiation dose in the first real angiographies of novice angiographers.

**Level of Evidence:**

Level 4, part 1: randomized trial, part 2: historically controlled study.

## Introduction

The need for neurointerventionalists performing thrombectomies has continued to increase in recent years. As at the same time quality and informative value of non-invasive imaging are improving, the decreasing number of diagnostic angiographies reduces the possibility of physicians to acquire angiographic skills. As an alternative training method, high-fidelity simulators have been successfully tested for validity in various fields in recent years [1–3]. Further studies demonstrated the ability of simulators to teach how to perform a real angiography and to improve a physician’s performance by repeated training [4, 5], but the proof of a direct benefit for an individual patient is still pending [5–7]. This would be, first, that the rate of direct complications is minimized. Those are mostly microemboli, producing (silent) cerebral ischemia. Second, radiation dose should be as low as possible. Third, the duration of the procedure should be short, as it correlates both with complications and radiation dose [8, 9].

To test the benefit of simulation training for a patient undergoing a diagnostic neuroangiography, we first analyzed the effect of a patient-anatomy-specific rehearsal on complication rate and compared the rate of silent cerebral ischemia in patients after diagnostic angiography with versus without prior patient-specific rehearsal. Second, we assessed the effect of a dedicated simulation curriculum for novice angiographers by comparing radiation exposure, duration, fluoroscopy time and amount of contrast agent of real angiographies of novice angiographers trained before the era of simulation versus novice angiographers trained with a dedicated simulation curriculum.

## Materials and Methods

### Patient-Specific Rehearsal

Patients who required a diagnostic angiography of all intracranial vessels in an elective setting were prospectively included from 21.06.2015 to 12.04.2017. They were randomized to the rehearsal and the control group. All patients underwent additional Magnetic Resonance Imaging (MRI) before {1–30 days} and the day after the angiography. The protocol always included an axial diffusion-weighted sequence (*b* = 1000 s/mm, TR 7637 ms, TE 55 ms, consecutive slices with an isotropic resolution of 2 mm; 3T, Achieva, Philips, NL). If there was no computed tomography angiography (CTA) or magnetic resonance angiography (MRA) including the aortic arch up to the skull base available, an MRA data set was acquired prior to the diagnostic angiography (coronal 3D gradient echo acquisition before and after intravenous injection of Gadolinium chelate, TR 5.9 ms, TE 1.92 ms). The number of DWI lesions was counted, and the difference between both scans was calculated. Since no DWI lesions were visible in the pre-scan, the difference matches with the number DWI lesions in the post-scan in every single case.

**Fig. 1:**
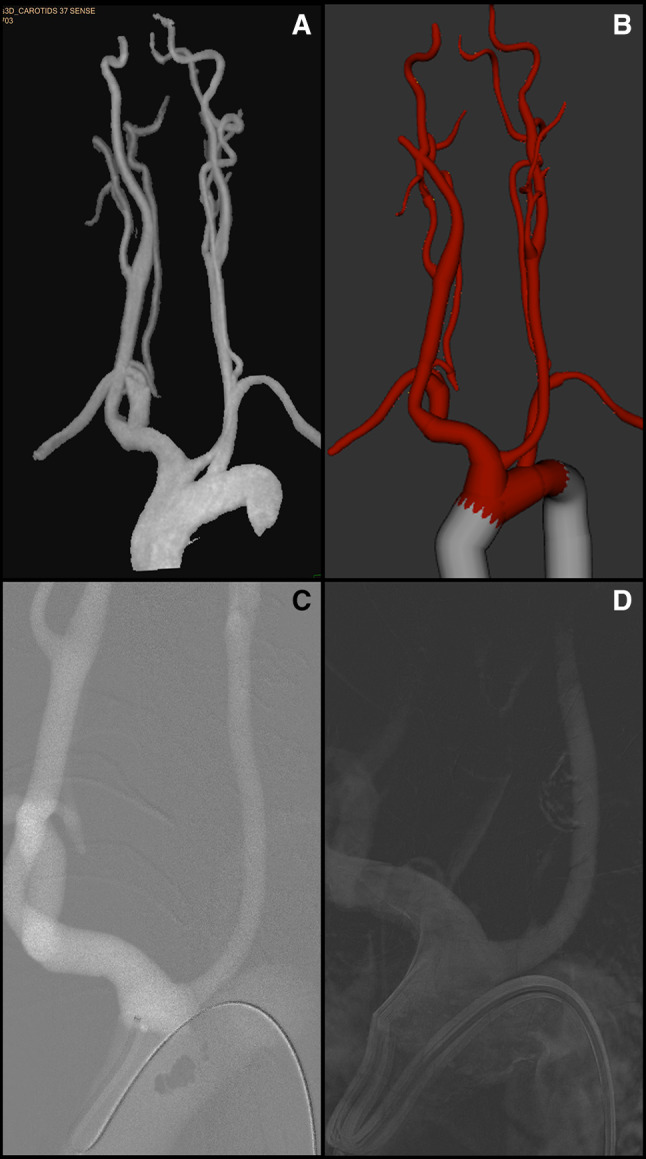
**A** semi-automatically segmented MRA, **B** anatomy after fusion with aortic template, **C** roadmap of aortic arch with sidewinder catheter during simulation, **D** roadmap of aortic arch with sidewinder catheter in real angiography

The vascular intervention system trainer VIST^®^ C (Mentice AB, SW) was used for this study. The stationary model VIST^®^ LAB consists of a control screen, two monitors for live biplane viewing, a monitor for displaying the series captured, a foot switch and a panel, e.g., for controlling the virtual C-arms. Pushing, pulling and rotating movements of the inserted wires and catheters are detected by three sensors able to provide haptic feedback. Air is injected instead of contrast medium. 3D CTA or MRA data were segmented semi-automatically (Fig. 1A) using IntelliSpace Portal (Philips, NL). The resulting 3D-Model was imported as an STL file to the software integrated in the simulator (Case-it, Mentice, SW). The model of the aortic arch with the supraaortic branches was connected to a template of the body’s trunk arteries (Fig. 1A), to be used for patient-specific rehearsal by the angiographer who later performed the real diagnostic angiography. An example of a roadmap in simulation and in a corresponding real angiography is shown in Fig. 1C, D. The physician who performed the real angiography simulated the case one day in advance.

During the real angiographies of all patients, the internal carotid artery and the vertebral artery were selectively probed on both sides and imaged in four planes. In some patients, one or both external carotid arteries were probed and imaged in two planes. 3D rotational series and target projections were supplemented if required. All catheters were continuously flushed with heparinized saline (2000 IU/1000 ml). All procedures were performed via transfemoral catheterization with a 5F sheath, standard guidewire (0.035″ Glidewire, Terumo, US) and 5F diagnostic catheters (Vertebral/Sidewinder I/Sidewinder II, Cordis, US). A non-ionic contrast medium (Imeron 300, Bracco, DE) was used for all procedures, manually injected.

For each patient, fluoroscopy time (FT), number of series, dose area product (DAP), numbers and shape of catheters and wires used were documented.

### Dedicated Simulation Curriculum

Retrospectively, the first 30 neuroangiographies of six novice angiographers were evaluated (n_total_ = 180). Three novices were taught conventionally by watching and gradually taking over supervised activities, until the novice was finally able to carry out a complete neuroangiography independently. The other three novices were trained by means of a structured simulation curriculum, before the conventional training started: After instruction in the operation of the simulator, the basic steps of a diagnostic neuroangiography were explained and demonstrated by an experienced angiographer. The first three simulations were performed accompanied by an experienced angiographer, and every fifth procedure was also supervised. In total, the novice angiographer must have completed at least 20 diagnostic panangiographies, before he was allowed to act on a real patient [1].

FT, amount of contrast, material used, DAP, number of series, number of images, number of vessels probed, and number of different catheters were retrospectively assessed in all 180 angiographies investigated.

Statistics: Due to the limited sample sizes, statistics are reported as median (interquartile range, IQR) using the mediantest. A p value ≤ 0.05 was considered significant. We performed all analyses using IBM SPSS Statistics, Version 23.0 (IBM, US).

Ethics: The local ethics committee approved the study (172/14) and all involved physicians and each patient in the patient-specific rehearsal study part provided informed consent before participating. The need for patient consent in the structured simulation curriculum study part was waived due to the retrospective nature of the study.

## Results

### Patient-Specific Rehearsal

We included 40 patients (median age 65.5, IQR 18, 14 males, 26 females, Table 1). Twenty patients without rehearsal of specific data prior to angiography (control group, median age 69.5, IQR 22, 8 male, 12 female) were compared to 20 patients with rehearsal (rehearsal group, median age 64, IQR 16, 6 male, 14 female). DWI lesions were found in 16 patients (40%), equally distributed to simulated and non-simulated patients. For details see Table 1. Overall, 32 DWI lesions occurred in the control group and 24 DWI lesions in the rehearsal group. In 10 cases, probing of one or more of the supraaortic branches did not succeed with the vertebral catheter and it was replaced by a sidewinder catheter. This happened in both groups (*n* = 4 in the non-simulated group, *n* = 6 in the simulated group) and was not associated with the occurrence of DWI lesions (mediantest *p* = 0.264). Due to various indications, cases differed in the number of vessels probed and imaged. Therefore, the calculation of total duration, FT, rounded amount of contrast and DAP, was additionally put into relation to the number of vessels displayed and to the number of series produced. None of the results showed a statistical difference (Table 2). Another factor influencing the data could be the expertise of the physician performing the angiography. Twenty-five angiographies were performed by a resident, 12 by a senior physician. In 3 angiographies, a senior physician took over because the resident had difficulty probing the vessels. Among those 3 there was one non-simulated case, which was the one with 19 DWI lesions, two simulated cases with none and 1 DWI lesion, respectively. Neither the comparison between non-simulated and simulated cases within a level of experience showed any difference, nor the comparison of residents with senior physicians (rehearsal group: 13 cases were performed by residents alone, 5 cases by senior physicians, and in 2 cases the catheters were handed over from the residents to a senior physician; control group: 12 cases were performed by residents alone, 7 cases by senior physicians, and in 1 case the catheters were handed over from the resident to a senior physician).

**Table 1:** Demographics of patients, data of angiographies and number of DWI lesions (median, Interquartile range, *p* values of mediantest < 0.05 are in bold type)

		Without rehearsal *n* = 20	With rehearsal *n* = 20	Mediantest
Age (years)	Median	69.5	64	0.752
	Interquartile range	22	16	
Total duration (min)	Median	30.0	32.5	1.000
	Interquartile range	19.5	27.25	
Fluoroscopy time (min)	Median	8.16	13.06	0.114
	Interquartile range	5.22	17.64	
Amount of contrast (ml)	Median	110	100	**0.043**
	Interquartile range	78	18	
Dose area product (mGy*cm^2^)	Median	95653	78227	0.343
	Interquartile range	20778	39334	
Number of series	Median	12	12	0.514
	Interquartile range	5	4	
DWI lesions	Median	0	0	0.747
	Interquartile range	1	2	
DWI lesions (number of patients)	Total	8	8	
	With 1 lesion	5	3	
	With 2 lesions	1	1	
	With 4 lesions	–	2	
	With 5 lesions	–	1	
	With 6 lesions	1	1	
	With 19 lesions	1	–

**Table 2:** Data of angiographies in relation of number of vessels probed and number of series (Median, Interquartile range, *p* values of mediantest < 0.05 are in bold type)

		Without rehearsal *n* = 20	With rehearsal *n* = 20	Mediantest
Total duration/imaged vessel (min)	Median	9.5	10.0	1.000
	Interquartile range	6.5	9.17	
Fluoroscopy time/imaged vessel (min)	Median	2.60	3.94	0.114
	Interquartile range	1.36	4.40	
Amount of contrast/imaged vessel (ml)	Median	32.00	28.33	0.340
	Interquartile range	13.88	9.58	
Dose area product/imaged vessel (mGy*cm^2^)	Median	27346	23532	0.343
	Interquartile range	12379	12822	
Total duration/serie (min)	Median	2.70	3.09	0.752
	Interquartile range	1.43	2.24	
Fluoroscopy time/serie (min)	Median	0.76	1.29	**0.027**
	Interquartile range	0.44	1.14	
Amount of contrast/serie (ml)	Median	9.30	8.33	0.114
	Interquartile range	4.63	3.04	
Dose area product/serie (mGy*cm^2^)	Median	7509	7416	0.752
	Interquartile range	3018	4718	

### Dedicated Simulation Curriculum

**Fig. 2:**
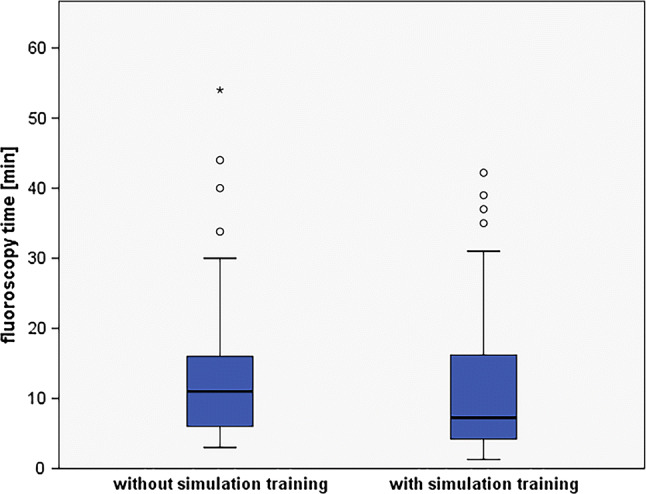
Median fluoroscopy time of 30 neuroangiographies of 6 novices, 3 without and 3 with prior simulation training. Boxplot, box limits: interquartile range, middle line: median, vertical lines: adjacent values (first quartile—1.5 IQR; third quartile  +  1.5 IQR), circles: outliers, asterix: extreme outliers

The group of 3 residents who underwent a structured simulation training before the first real procedure showed significantly shorter median FT than 3 residents who had not received training (FT median 7.25 min/IQR 12 vs. 11 min/IQR 10, Fig. 2) [1]. DAP was almost 20% lower in the simulation trained group (45,540 mGycm^2^/IQR 53916 vs. 55488 mGycm^2^/IQR 60697) [1], but this difference did not reach statistical significance. Alike, differences both in the total number of images (171.5/IQR210 vs. 207/IQR 248) and in the number of images per series (24/IQR 16.25 vs. 27/IQR 20) did not reach statistical significance. Other parameters like the amount of contrast, number of series, number of vessels probed, and number of catheters showed no difference (Table 3).

**Table 3:** 30 angiographies of 6 novices, 3 without and 3 with prior simulation training (Median, Interquartile range, *p* values of mediantest < 0.05 are in bold type)

		Without simulation training	With simulation training	Mediantest
Fluoroscopy time (min)	Median	11	7,25	**0.025**
	Interquartile range	10	12	
Amount of contrast (ml)	Median	100	100	0.351
	Interquartile range	20	20	
Dose area product (mGy*cm^2^)	Median	55488	45540	0.655
	Interquartile range	60697	53916	
Number of series	Median	7	7	0.654
	Interquartile range	8	6	
Number of images	Median	207	171.5	0.180
	Interquartile range	248	210	
Number of vessels	Median	2	2	0.764
	Interquartile range	3	2	
Number of different catheters	Median	1	1	0.106
	Interquartile range	0	0	
Average DAP/serie (mGy*cm^2^)	Median	6952	7402	0.456
	Interquartile range	4605	3500	
Number of images/serie	Median	27	24	0.296
	Interquartile range	20	16.25	
Dose area product/imaged vessels (mGy*cm^2^)	Median	21575	24650	0.297
	Interquartile range	13208	12737	

## Discussion

In this study, we were not able to show an effect of patient-specific rehearsal of diagnostic neuroangiographies on direct complications or quality measures of the subsequent angiography. In contrast, we were able to show that a structured simulation curriculum increased the performance of novice angiographers in real angiographies.

Clinically relevant complications occur rarely in neuroangiographies, especially in diagnostics. The rate of major complications is reported as about 2% and only a few are associated with permanent neurological deficits [10]. However, it is known that a considerable number of diagnostic angiographies lead to silent cerebral ischemia which are not noticed by the patient. Various authors report about 9 to 29% [8, 9, 11, 12]. Whether a small cerebral ischemia is asymptomatic or not cannot be influenced. Even a very small lesion can cause serious deficits if it occurs in an eloquent area. Hence, it can be assumed that a reduction in the rate of these lesions would also lead to a reduction in the rate of real complications. In our cohort, however, there was no difference between the non-simulated and the simulated cases.

It is suspected that these lesions are caused by microembolization of air, thrombi or the scraping off of atherosclerotic plaques. By means of Doppler sonography, it has been shown that 90% of microemboli occur during injections and only 10% are conditioned by catheter and wire manipulation [13]. It is therefore not surprising that the number of patients with DWI lesions was similar in both groups. The amount of contrast agent was in fact lower in simulated patients. However, the total amount of injected contrast agent can never be recorded precisely, when manual injections are performed with unrecorded amounts of contrast remaining in the syringe after the injection. Therefore, a difference of 10 ml should not be overvalued.

The third aspect, the scraping off of atherosclerotic plaques, is said to be associated with vascular risk diseases. Although some patients in our cohort suffered from hypertension, diabetes mellitus or coronary heart disease, this rate was not higher among those with post-angiographic DWI lesions.

Assumedly, a careful analysis of the specific anatomy, which inevitably accompanies each patient-specific rehearsal, could lead to fewer atherosclerotic plaques being scraped off in reality. However, this would only be possible if these plaques could be perceived during the rehearsal and consciously avoided in reality. In the segmented data used for simulation, only the lumina of the vessels are represented, plaques are not recognizable. It is presumed that the use of several catheters leads to an increase of DWI lesions [11, 12]. In our cohort, an additional catheter was needed in 25% of cases, but did not affect the rate of DWI lesions.

It was noteworthy that an overall rate of 40% patients with DWI lesions was almost twice as high as reported in the literature. Although the average age was also lower (42.9–58.25 years) than in our cohort (66.25), it is more likely to be due to the technical progress in MR imaging. In all former studies, DWI was acquired in 5-mm-thick slices at 1.5T, while in our study, 2-mm-thin slices were performed at 3T.

It has rarely been tested whether the effect of simulation training is reflected in improved performance in reality, i.e., faster completion, lower radiation dose or less amount of contrast agent. Among interventional cardiologists, a two-day course with 6 h of practical simulation had no effect on the real performance [14]. Later, the same group could prove that a 10-hour training for senior residents led to a significantly shorter fluoroscopy time and shorter treatment times—shown in only two subsequent angiographies [15]. Furthermore, the transfer into reality was successful for iliac interventions in a pig model [16] and in cardiac transseptal punctures [17]. Our evaluation of 3 residents without versus 3 residents with completed simulation curriculum compared a somewhat larger number of 30 neuroangiographies. Here, a significant reduction in fluoroscopy time and a reduction in DAP of 20% was shown. The total duration of procedures was not reduced, a parameter that turned out to be an indirect predictor for a lower complication rate in previous studies [8, 18–20].

Overall, it remains difficult to prove the direct benefit of simulation training for an individual patient. One reason might be that human factors may have a greater impact on patient`s benefits than the potential of acquiring manual skills during simulation [21]. Our preliminary perception reveals that novices can behave much more professionally after completing the simulation curriculum. They have to focus less on the operation of the angiosuite or the basic handling of catheters. In return, they are more capable to react to the individual patient anatomy or to detect pathology already during the image acquisition. Thus, simulation training should play a more important role in training, especially for beginners. Before the first contact with a real patient, many manipulations can be practiced, and methodical considerations can and should be made. Additionally, there is the possibility to repeat numerous details of a real angiography in a safe environment during the first basic training in reality. Considering the results of this study, currently available simulators may not play a role for advanced angiographers or interventionalists. If the quality of interventional simulations, such as in aneurysm therapy, is further improved, even an advanced neurointerventionalist could benefit from simulation (training). Whether a simulation immediately preceding a treatment can prove itself—e.g., in thrombectomy—will also depend on the speed of data segmentation and the realistic implementation of state of the art devices in the simulator [1].

## Limitations

In patient-specific rehearsals, many different physicians were involved (9 residents and 5 senior physicians), due to the organizational structures of everyday clinical practice. Thus, on the one hand, numerous influencing factors have to be taken into account (e.g., individual talent), but on the other hand, the normal clinical situation is depicted quite realistically. In contrast, the second study part only involved 3 residents per training group. Due to the modernization of the angiosuite, the subsequent examination data, with regard to radiation dose, fluoroscopy time and total duration, would no longer have been comparable with earlier examinations in more novice angiographers, limiting these numbers. Moreover, it may not have been documented in every case, if, when and how a senior physician intervened in an angiography, which was assigned to a resident. As an example, a two-second correction of the catheter movement in the right moment by the supervising senior physician might have been not be documented but changed the procedure times considerably.

Both parts of the study were carried out in a high-volume center with sufficient staff, allowing for high-quality angiographic training even before using simulators. In a low-volume center with staffing shortage, one could expect even greater differences between the conventional training path and training with simulation. A high level of expertise might also have influenced the negative results of the patient-specific rehearsal.

## Conclusion

For an unselected patient cohort, patient-specific rehearsal had no measurable benefit.

Further studies are needed to prove whether patients with particularly difficult anatomies could still benefit and if this benefit is similar in all experience levels of angiographers.

A structured simulation-based curriculum for teaching angiographic skills results in a reduction of fluoroscopy time and radiation dose and thus should be included in the initial teaching curriculum for all novice angiographers.
